# Leptospirosis in horses: Sentinels for a neglected zoonosis? A systematic review

**DOI:** 10.14202/vetworld.2023.2110-2119

**Published:** 2023-10-14

**Authors:** Eduardo A. Díaz, Gabriela Arroyo, Carolina Sáenz, Luis Mena, Verónica Barragán

**Affiliations:** 1 Escuela de Medicina Veterinaria, Colegio de Ciencias de la Salud, Universidad San Francisco de Quito, Diego Robles, Quito, 170157, Ecuador; 2 Hospital de Fauna Silvestre Tueri, Instituto iBIOTROP, Universidad San Francisco de Quito, Diego Robles, Quito, 170157, Ecuador; 3 Carrera de Medicina Veterinaria, Facultad de Ciencias Pecuarias, Escuela Superior Politécnica de Chimborazo ESPOCH, Riobamba, 060155, Ecuador; 4 Colegio de Ciencias Biológicas y Ambientales, Instituto de Microbiología, Universidad San Francisco de Quito, Diego Robles, Quito, 170157, Ecuador

**Keywords:** Ecuador, equine leptospirosis, neglected tropical zoonosis, one-health, sentinel species, systematic review

## Abstract

**Background and Aim:**

Leptospirosis is considered a neglected tropical zoonosis in low-income countries due to surveillance system limitations and non-specificity of symptoms. Humans become infected through direct contact with carrier animals or indirectly through Leptospira-contaminated environments. Conventionally, equines have been considered an uncommon source of leptospirosis, but recent publications in Latin America suggest that their role in the maintenance and dispersion of the bacteria could be more relevant than expected, as horses are susceptible to a wide variety of zoonotic Leptospira spp. from domestic and wild animals with which they share the environment. A systematic review of the published literature was conducted to compile the available information on Leptospira spp. in Ecuador, with a special focus on equine leptospirosis, to better understand the epidemiology of the bacterium and identify possible knowledge gaps.

**Materials and Methods:**

A systematic review of the published literature was conducted in PubMed, SciELO and Web of Science databases to compile the available information on Leptospira spp. in Ecuador, with a special focus on equine leptospirosis, to better understand the epidemiology of the bacterium. We used a combination of the terms (Leptospira OR Leptospirosis) AND Ecuador, without restrictions on language or publication date.

**Results:**

Our literature review reveals that published scientific information is very scarce. Eighteen full-text original scientific articles related to Leptospira or leptospirosis cases in Ecuador were included in the systematic review. Most of the studies reported data obtained from one of the four regions (Coast), and specifically from only one of the 24 Provinces of Ecuador (Manabí), which evidence a large information bias at the geographical level. Furthermore, only the studies focused on humans included clinical signs of leptospirosis and there is only one study that analyzes the presence of Leptospira spp. in water or soil as a risk factor for pathogen transmission. Finally, only one study investigated Leptospira in horses.

**Conclusion:**

Since sentinel species can provide useful data on infectious diseases when epidemiologic al information is lacking, and horses could be considered excellent sentinel species to reveal circulating serovars, we propose developing a nationwide surveillance system using horses. This cost-effective epidemiological survey method provides a baseline for implementing specific prevention and control programs in Ecuador and neighboring developing countries.

## Introduction

Leptospirosis is a re-emerging zoonosis of public health concern due to morbidity and mortality in both humans and animals [1]. Humans are commonly infected through direct contact with the urine of *Leptospira* spp. carrier animals or indirectly by contaminated environments with this pathogen [2]. The disease is predominantly distributed among low-income populations in tropical developing countries [3]. It is estimated to cause more than 1 million severe human cases and approximately 60,000 deaths/year. However, the limitations of surveillance systems in impoverished regions probably contribute to an underestimation of the real impact of leptospirosis [4]. Furthermore, leptospirosis is usually underdiagnosed because of the difficulty in distinguishing its clinical signs from those of other endemic febrile diseases [5]. An overview of risk factors for leptospirosis suggests that epidemiological patterns are closely related to the bioclimatic context with heavy seasonal rains in tropical countries being one of the main risk factors [6]. Climate change and extreme weather events such as cyclones and floods are expected to occur with greater frequency and intensity, which could lead to increased leptospirosis outbreaks [7].

Latin America has one of the highest estimated incidences of leptospirosis in the world, including regions where the burden of the disease is underappreciated [4, 8]. Usually, health authorities report cases that occur after floods in urban areas, but the cases in rural areas often go unreported [9]. In Ecuador, extreme weather conditions, such as “El Niño”, and socio-economic factors, such as poor sewage infrastructure and inadequate hygienic conditions in rural areas, contribute to the fact that leptospirosis continues to be a neglected growing problem [10, 11]. Indeed, Chiriboga *et al*. [12] detected *Leptospira*-DNA in a high percentage of febrile patients from rural communities who had been tested for several endemic diseases, but not for leptospirosis. Barragan *et al*. [13] also found *Leptospira*-DNA in febrile people, cattle, and pigs from rural communities, suggesting that these animals may be the most important reservoir for human transmission. Nevertheless, recent bibliographic reviews have shown that equine leptospirosis may be more common than expected in Latin America [14, 15]. Leptospirosis in horses has traditionally been considered relatively uncommon, but new data suggest that the infection is widespread, with an incidence and infecting serovars varying considerably between different geographical regions [16]. Management factors such as the presence of other animal species, increasing age and herd size, free-ranging feeding practices, drinking untreated water, flooding, and poor sanitation are associated with increased risk of exposure to *Leptospira* spp. in horses [17–22]. Clinical signs include reproductive disorders, renal and hepatic dysfunctions, respiratory distress, and recurrent uveitis, but most infections remain asymptomatic [23]. Several worldwide studies have shown that, although the prevalence of infection may be higher than for other species, unrecognized subclinical infections commonly occur in apparently healthy horses [18, 24–28]. However, infected horses can become carriers and contribute to maintaining the bacterium in the environment by shedding *Leptospira* spp. in their urine [27, 29]. Seroprevalence and isolation studies indicate that the horse is susceptible to various incidental infections from different serovars [23, 30, 31]. Accordingly, since humans and other animals exposed to the bacteria share the environment with horses, these could be considered excellent sentinel species to reveal circulating serovars [20, 32, 33]. Serovars present in tropical regions with high wildlife richness are generally related to the wide range of mammalian reservoirs because wild mammals are more likely to be infected with *Leptospira* spp. [34, 35]. Ecuador is one of the most megadiverse countries on the planet, with hundreds of registered mammal species [36, 37]. This biodiversity, added to the climatic and socio-economic conditions of the region, makes it an ideal place to study the complex epidemiology of *Leptospira* spp. [13].

Equine population has increased considerably in recent years in Ecuador, both in peri-urban and rural areas for recreational activities and in more remote areas where they continue to be essential for agricultural labors [38]. Since the use of sentinel species may be a cost-effective study target to improve epidemiological surveillance in developing countries [39, 40], and horses have proven to be a suitable species for revealing circulating leptospiral serovars [20, 32, 33], we propose their use as sentinel species in the region.

A systematic review of the published literature was conducted to compile the available information on *Leptospira* spp. in Ecuador, with a special focus on equine leptospirosis, to better understand the epidemiology of the bacterium and identify possible knowledge gaps. Our results will improve prevention and control programs for both humans and animals.

## Materials and Methods

### Ethical approval

This study does not require ethical approval. Following Preferred Reporting Items for Systematic Reviews and Meta-analyses guidelines, we conducted a systematic review to collect current knowledge on *Leptospira* in Ecuador.

### Study period and location

This study was conducted from January to May 2023 at the Universidad San Francisco de Quito, Diego Robles, Quito, 170157, Ecuador.

### Search strategy

Published literature was searched in PubMed (http://www.ncbi.nlm.nih.gov/pubmed), SciELO (https://scielo.org) and Web of Science (http://apps.webofknowledge.com) databases on April 20, 2023, without restrictions on language or publication date. We used a combination of the terms (Leptospira OR Leptospirosis) AND Ecuador.

### Article selection process

After retrieving the results from the databases, duplicate articles were identified and discarded by sorting the titles alphabetically using an Excel 2010 spreadsheet. At least two researchers read each article to confirm its relevance to the scope of the review. If a disagreement was observed, a third researcher was involved to further include or exclude the article. Article triage was performed in three stages, involving review of article’s title, abstract, and full-text in accordance with inclusion and exclusion criteria.

### Inclusion and exclusion criteria

Eligibility criteria included full-text original scientific articles related to *Leptospira* or leptospirosis in Ecuador; abstracts, letters to the editor, and articles without original data or non-scientific communications were excluded from the screening process.

### Data extraction and synthesis

The following information was extracted from each study that met the inclusion criteria: (a) Article reference, (b) study area, (c) study species, (d) type sample, (e) positivity, (f) clinical signs, (g) *Leptospira* species, and (h) *Leptospira* serovars.

## Results

**Figure-1:**
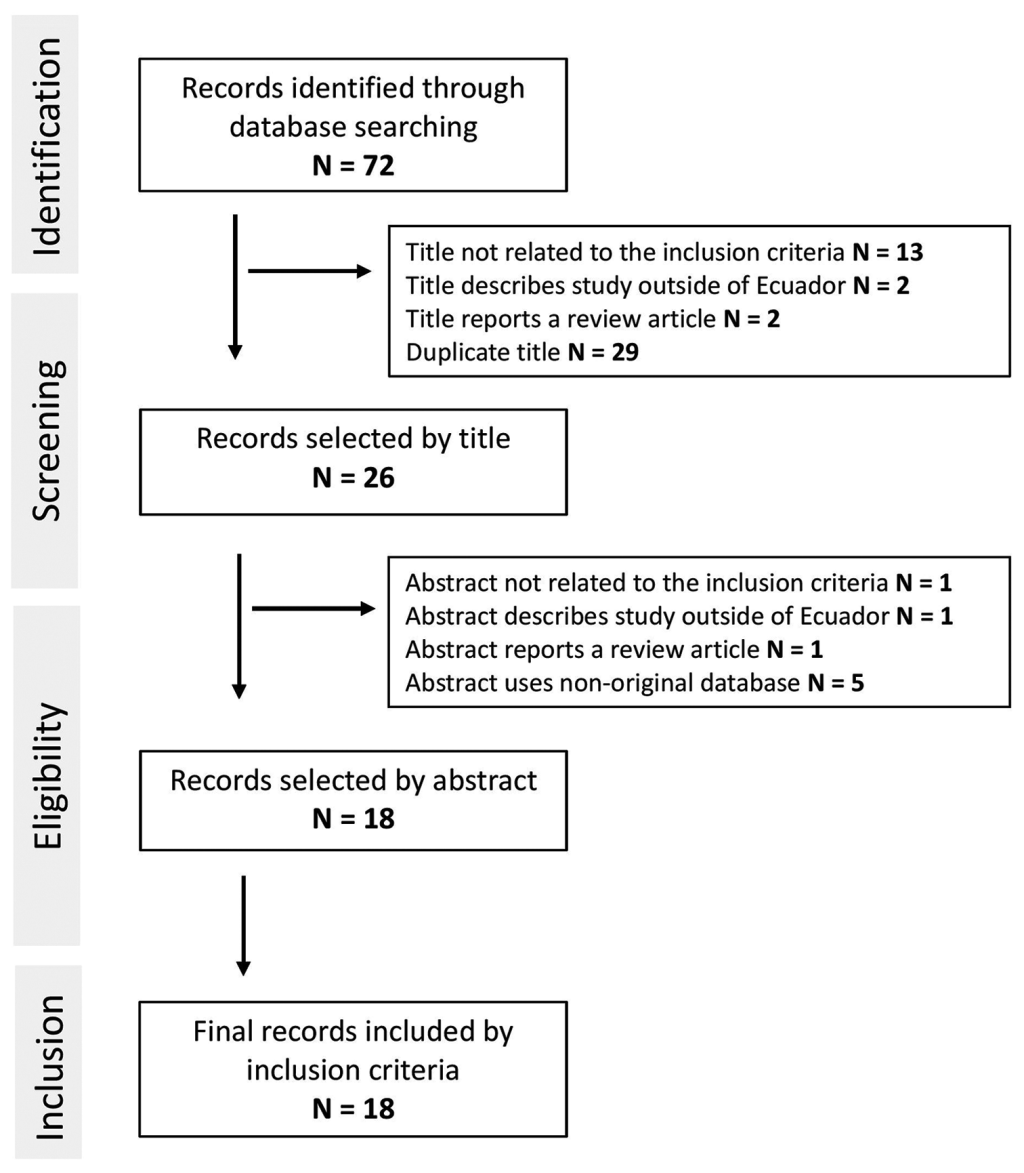
Flow diagram of the study selection process and exclusion identified.

A total of 72 articles were retrieved according to the search terms (6 from SciELO, and 31 from Web of Science, and 35 articles from PubMed). After removing 29 duplicate articles, 43 articles were screened by title. Of these, 13 were excluded because they were not related to the inclusion criteria, two described studies outside of Ecuador, and two reported review articles. By screening the remaining 26 abstracts, one was excluded as it was not related to the inclusion criteria, one described a study outside of Ecuador, one reported a review article, and five used a non-original database. Finally, 18 full-text original scientific articles related to *Leptospira* or leptospirosis cases in Ecuador were included in the systematic review. The flow diagram of the search strategy is summarized in Figure-1.

**Figure-2:**
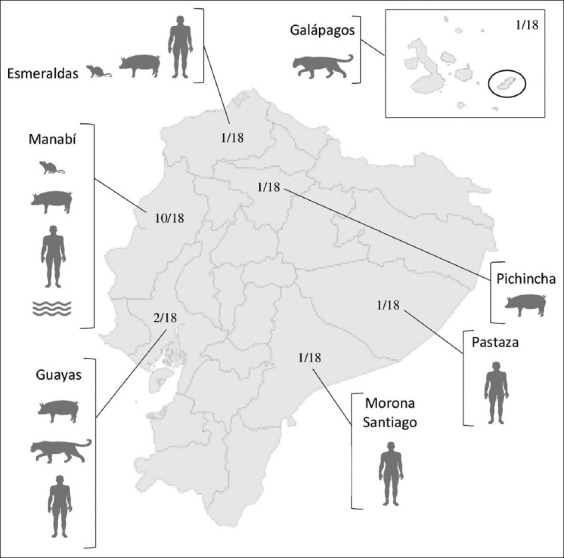
Provinces with published on Leprosaria in humans 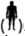, synanthropic 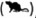, domestic 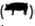, wild 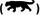 species and/or the environment 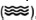. The first number shows the research from each province, the second number is the total of research identified in Ecuador (one study did not specify location). NO research was found in Azuay, Bolivar, Cañar, Carchi, Chimborazo, Cotopaxi, El Oro, Imbabura, Loja, Los Rios, Napo, Orellana, Santa Domingo, Sucumbíos, Tungurahua, and Zamora.

The number of published studies investigating *Leptospira* or leptospirosis in Ecuador increased over the years and more significantly in the last decade (before 2002, n = 0; between 2002 and 2011, n = 3; between 2012 and 2021, n = 15). Articles reported data obtained from three geographical regions (Coast n = 13; Amazon n = 2; Andes n = 1; and Galapagos n = 1) and six provinces (Manabí n = 10; Guayas n = 2; Esmeraldas n = 1; Galapagos n = 1; Morona Santiago n = 1; Pastaza n = 1; and Pichincha n = 1); one study did not specify the geographical region or province. Included studies were mostly focused on domestic animals (n = 7) followed by those involving humans (n = 5), while fewer studies were centered on wildlife (n = 1) and *Leptospira* spp. in the environment (n = 1). Regarding the interrelationship between humans, domestic animals, wildlife, and the environment, the search retrieved studies that included information on humans, domestic and synanthropic species (n = 2), humans and domestic species (n = 1), and domestic and wild species (n = 1); no research provided joint data on all of them. The geographical location and studied populations are summarized in Figure-2.

In total, 1638 people and at least 15 different animal species were tested, including seven domestic species: 2192 cows (*Bos taurus*), 639 pigs (*Sus domesticus*), 34 dogs (*Canis familiaris*), three European rabbits (*Oryctolagus cuniculus*), three horses (*Equus caballus*), three sheep (*Ovis aries*), and one Guinea pig (*Cavia porcellus*); seven wild species: Seven Galapagos sea lions (*Zalophus wollebaeki*), three lions (*Panthera leo*), two ring-tailed coatis (*Nasua nasua*), one common woolly monkey (*Lagothrix lagotricha*), one Ecuadorian white-fronted capuchin (*Cebus aequatorialis*), one mountain coati (*Nasuella olivacea*), and one oncilla (*Leopardus tigrinus*); and one synanthropic genus: 107 rats (*Rattus* spp.). Regarding the presence of *Leptospira* in the environment, 133 soil samples and 136 river water samples were analyzed.

Most of the studies used serological techniques to detect exposure (n = 11), while only three studies identified *Leptospira* with molecular methods, allowing the identification of the infecting species. A total of 16 different serovars (Australis, Autumnalis, Ballum, Bataviae, Bratislava, Canicola, Cophenhageni, Cynopteri, Gripotyphosa, Hardjo, Icterohaemorrhagiae, Pomona, Pyrogenes, Sejroe, Tarassovi, and Wolffi) were identified by microscopic agglutination test (MAT). The most common serovars were Canicola and Icterohaemorrhagiae, which were detected in seven of the eight studies that provided serovar information. A total of eight *Leptospira* species were identified (*L. borgpetersenii*, *L. inadai*, *L. interrogans*, *L. kirschnerii*, *L. licerasiae*, *L. noguchii*, *L. santarosai*, and *L. wolffii*). Reported positivity ranged from three to 100%, being above 50% in 10 of the 16 studies that provided this information. Interestingly, although there are only published data from three horses, all of them were seropositive, and it was the species that presented titers ≥1:200 for more different serovars of all the species investigated. Finally, almost all human studies (n = 7/8) included clinical signs (mainly fever). In contrast, no animal studies included clinical signs and only two associated the presence of *Leptospira* antibodies with kidney lesions confirmed by necropsy. Information extracted from the studies that met the inclusion criteria is summarized in Table-1 [12, 13, 41–56].

**Table-1:** Selected studies about *Leptospira* findings and leptospirosis cases in Ecuador.

Region (province)	Species (N)	Sample (Assay*)	Positive (%)	Clinical signs	*Leptospira*species**	*Leptospira*serovars***	References
Coast (Esmeraldas)	*B. taurus*(27)	Urine (PCR)	74	-	1, 2, 4, 8	-	[12]
	*C. familiaris*(30)	Urine (PCR)	70	-	2	-	
	*H. sapiens*(464)	Blood (PCR)	40.7	-	1, 2, 8	-	
	*Rattus*spp. (6)	Kidney (PCR)	100	-	1, 2, 4, 8	-	
	*S. domesticus*(27)	Urine (PCR)	66.6	-	1, 2	-	
Coast (Manabí)	*C. familiaris*(30)	Urine (PCR)	70	-	2	-	[13]
	*H. sapiens*(464) *Rattus*spp. (6)	Blood (PCR)	40.7	Fever	1, 2, 8	-	
		Kidney (PCR)	100	-	1, 2, 4, 8	-	
	*S. domesticus*(27)	Urine (PCR)	66.6	-	1, 2	-	
	*S. domesticus*(128)	Urine (PCR)	21.1	-	1, 3, 5, 7, 8	-	
Galapagos (Galapagos)	*Z. wollebaeki*(7)	Kidney/Placenta (PCR)	71.4		*Leptospira*spp.	-	[41]
Coast (Guayas)	Water (136)	Water (PCR)	3.6	-	*Leptospira*spp.		[42]
	*C. porcellus*(1)	Blood (MAT)	100	-	-	4, 11	
	*C. familiaris*(4)	Blood (MAT)	100	-	-	6, 9, 10, 11	
	*C. aequatorialis*(1)	Blood (MAT)	100	-	-	4, 6, 7, 8, 10	
	*E. caballus*(3)	Blood (MAT)	100	-	-	1, 2, 4, 6, 7, 8, 9, 10, 11, 12, 14, 15	
	*L. lagotrichia*(1)	Blood (MAT)	100	-	-	4, 6, 8, 9, 10, 11, 16	
	*L. tigrinus*(1)	Blood (MAT)	100	-	-	2, 6, 7, 8, 10, 11, 12, 14, 16	
	*N. nasua*(2)	Blood (MAT)	100	-	-	1, 2, 4, 6, 7, 9, 10, 11, 12, 14, 16	
	*N. olivacea*(1)	Blood (MAT)	100	-	-	2, 6, 7, 10	
	*O. cuniculus*(3)	Blood (MAT)	100	-	-	4, 6, 9, 10, 11	
	*O. aries*(3)	Blood (MAT)	100	-	-	6, 9, 10, 11	
	*P. leo*(3)	Blood (MAT)	100	-	-	6, 7, 8, 10, 12, 14	
	*S. domesticus*(3)	Blood (MAT)	100	-	-	4, 6, 10	
Andes (Pichincha)	*S. domesticus*(1)	Urine (PCR)	0	-	-	-	[43]
		Sperm (PCR)	33	-	7	-	
		Post-sperm (PCR)	100	-	7	-	
		Kidney (PCR)	100	-	7	-	
		Epididymis (PCR)	0	-	-	-	
		Testicles (PCR)	100	-	7	-	
Coast (Manabí)	*B. taurus*(2)	Urine (GS)	-	-	3	-	[44]
	*H. sapiens*(1)	Blood (GS)	-	Fever	7	-	
Coast (Manabí)	*B. taurus*(320)	Blood (MAT)	50.9	kidney lesions	-	5, 6, 7, 9, 10, 11, 12, 16	[45]
Coast (Guayas)	*H. sapiens*(1)	Blood (MAT)	100	Fever	-	3, 11	[46]
-	*H. sapiens*(2)	Blood (Culture)	100	Fever	*Leptospira*spp.	-	[47]
Coast (Manabí)	*B. taurus*(854)	Blood (MAT)	57.4	-	-	5, 6, 7, 9, 10, 11, 12, 16	[48]
Amazon (Pastaza)	*H. sapiens*(272)	Blood (ELISA)	14.7	Fever	-	-	[49]
Coast (Manabí)	Soil (133)	Soil (PCR)	24	-	*Leptospira*	-	[50]
	Water (136)	Water (PCR)	3.6	-	spp.*Leptospira*spp.		
Coast (Manabí)	*B. taurus*(72)	Urine (PCR)	13.8	-	-	-	[51]
Amazon (Morona)	*H. sapiens*(216)	Blood (ELISA)	50	-	-	-	[52]
Coast (Manabí)	*B. taurus*(749)	Blood (MAT)	56.2	-	-	5, 6, 7, 9, 10, 11, 12, 16	[53]
Coast (Manabí)	*H. sapiens*(2)	Blood (ELISA)	100	Fever	-	-	[54]
Coast (Manabí)	*S. domesticus*(280)	Blood (MAT)	18.9	-	-	1, 4, 6, 10, 11, 14, 15, 16	[55]
Coast (Manabí)	*S. domesticus*(200)	Blood (MAT)	16.5	Kidney lesions	-	1, 5, 6, 9, 11, 12, 13, 15	[56]

* Assay: ELISA=Enzyme linked immunosorbent assay, GS=Genome sequencing, HE=Hematoxylin-Eosin, MAT=Microscopic agglutination test, ME=Macroscopic examination, PCR=Polymerase chain reaction;

** *Leptospira* species: 1=*L. borgpetersenii*, 2=*L. inadai*, 3=*L. interrogans*, 4=*L kirschnerii*, 5=*L. licerasiae*, 6=*L. noguchii*, 7=*L. santarosai*, and 8=*L. wolffii*;

*** Leptospira serovars: 1=Australis, 2=Autumnalis, 3=Ballum, 4=Bataviae, 5=Bratislava, 6=Canicola, 7=Copenhageni, 8=Cynopteri, 9=Grippotyphosa, 10=Hardjo, 11=Icterohaemorragiae, 12=Pomona, 13=Pyrogenes, 14=Sejroe, 15=Tarassovi, and 16=Wolffi. *B. taurus*=*Bos taurus, C. familiaris*=*Canis familiaris, H. sapiens*=*Homo sapiens, S. domesticus*=*Sus domesticus, Z. wollebaeki*=*Zalophus wollebaeki,C. porcellus*=*Cavia porcellus, C. aequatorialis*=*Cebus aequatorialis, E. caballus*=*Equus caballus, L. lagotrichia*=*Lagothrix lagotricha, L. tigrinus*=*Lentinus tigrinus, N. nasua*=*Nasua nasua, N. olivacea*=*Nasuella olivacea,O. cuniculus*=*Oryctolagus cuniculus, O. aries*=*Ovis aries, P. leo*=*Panthera leo.*

## Discussion

A recent epidemiological analysis of regional data showed that leptospirosis is a major public health problem in Latin America and that Ecuador is among the countries with the highest incidence rate per capita. However, the authors also highlight that not all regions have robust surveillance systems, and that more animal studies are needed to better understand the current epidemiological situation [9]. In fact, the latest reviews on animal leptospirosis in Latin America show no records from Ecuador [35, 57]. Our systematic review is the first to compile and analyze data on the human, animal, and environmental epidemiology of *Leptospira*, focusing on horses as possible sentinel species.

We found that most of the studies reported data obtained from one of the four regions (Coast), and specifically from only one of the 24 Provinces of Ecuador (Manabí), which evidence a large information bias at the geographical level. Furthermore, only the studies focused on humans included clinical signs of leptospirosis and, although humans and animals may be exposed to the pathogen through contact with a contaminated environment, there is only one study that analyzed the presence of *Leptospira* spp. in water or soil as a risk factor for pathogen transmission. A one-health approach, which integrates the human-animal-environment interface, is the ideal framework to better understand and fight leptospirosis. Prevention and control measures should be approached from this perspective, but a major limiting factor has been the lack of communication and cooperation between the human and animal health-care communities [35, 58, 59]. In Ecuador, human leptospirosis is considered by the Epidemiology Department of the Ministry of Public Health a notifiable disease, with an annual incidence ranging between 0.27 and 2.45 cases/100,000 inhabitants, and a fatality rate of 3.06% [60]. However, to the best of our knowledge, there is no government data on morbidity and mortality patterns in domestic or wild animals.

Direct and indirect evidence suggest that leptospirosis is largely widespread among wildlife in Latin America [35, 57]. Surprisingly, our review shows that only two studies included samples from wild mammals. Denkinger *et al*. [41] found pathogenic *Leptospira* spp. in 71.4% of Galapagos sea lions, and Orlando *et al*. [42] found 100% seropositivity in common woolly monkeys, Ecuadorian white-fronted capuchin, Lion, Mountain coati, Oncilla, and Ring-tailed coati. In addition, two studies [12, 13] included synanthropic rodents of the genus *Rattus*, showing a positivity of 3% and 100%, respectively. The small number of studies on wild species is alarming and requires special attention, given that the role of different animal hosts remains unclear. Something similar occurs with domestic species, since although ten studies that included samples of domestic animals, eight of these specifically studied cattle and/or pigs. In addition, there was a great variation in the number of individuals evaluated between species, with only three animals studied in the case of horses to more than 2000 cows. Therefore, our findings reflect the scarce information on leptospirosis in domestic animals in the region.

*Leptospira’*s capacity to infect multiple hosts creates surveillance challenges but also provides opportunities to collect data from animal species that can be used to detect risks to human health. In developing countries, where data on the epidemiology of infectious agents is still lacking, the use of sentinel species can provide essential disease baseline data while enhancing cost-effective surveillance [39, 40]. Members of the order Carnivora are predators and/or scavengers that feed on a wide range of species, exposing themselves to various pathogens. Wild and domestic carnivores (especially dogs) have been proposed as good sentinels for epidemiological surveillance of leptospirosis [60–66]. In Ecuador, most wild carnivores are listed as threatened [67], which limits sampling opportunities due to regulatory and logistical constraints. Regarding domestic carnivores, there are vaccines against leptospirosis authorized for use in dogs, and the vaccination status may alter the serological results of the inoculated animals [68, 69]. In addition, there is no updated national registry of domestic dogs, which makes the epidemiological interpretation of results even more difficult. However, no vaccines against Leptospira are authorized for use in horses, and a national equine registry would allow longitudinal studies to be conducted to evaluate changes in the epidemiology of the disease. Serological surveys in many countries worldwide have shown that horses are exposed to a wide range of serovars, inferring complex epidemiology depending on climatic conditions and the presence of maintenance hosts. For example, 15 different serovars were detected in Switzerland [18], 16 in Ethiopia [20], 12 in Korea [24], 21 in South Africa [31], 20 in Australia [32], 16 in Brazil [70] and 17 in Poland [71]. Therefore, we agree with previous studies by Tsegay *et al*. [20], Wangdi *et al*. [32], and Dewes *et al*. [33] that horses could be a useful sentinel species to assess the occurrence of *Leptospira* regionally, allowing their use in Ecuador to avoid sampling bias and possible errors in the interpretation of results.

In our review, only Orlando *et al*. [42] investigated the seroprevalence of *Leptospira* in three horses and detected anti-*Leptospira* antibodies in all the specimens tested. The panel of antigens used for the MAT contained 21 serovars representing six *Leptospira* species (*L. borgpetersenii* serovars: Castellonis, Javanica, Tarassovi; *L. interrogans* serovars: Australis, Autumnalis, Bataviae, Bratislava, Canicola, Copenhageni, Hardjo, Hebdomadis, Pomona, Pyrogenes, Icterohaemorrhagiae, and Wolfii, Sejroe; *L. kirschneri* serovars: Cynopteri, Grippotyphosa; *L. noguchii* serovar: Panama; *L. santarosai* serovars: Shermani; and *L. biflexa* serovar: Patoc). Of the 21 serovars used, 12 were detected in hoses. The major serovars, in order of their decreasing seroprevalence, were Bataviae (100%), Gripotyphosa (100%), Icterohaemorrhagiae (100%), Autumnalis (67%), Canicola (67%), Cynopteri (67%), Hardjo (67%), Australis (33%), Cophenhageni (33%), Pomona (33%), Sejroe (33%), and Tarassovi (33%). All three horses presented titers ≥1:200 for more than one serovar. This study also included sera from seven domestic and 6 wild species. Although the low number of horses tested does not allow for conclusive considerations, the species presented reactivity to more different serovars. Serologic reactions to multiple serovars are common in horses; the reasons why a serum reacts with several serovars may be a cross-reaction or a coinfection with more than one serovar [18, 24]. The serovar providing but the highest antibody titer could be an infecting serovar. However, all serovars showing an antibody titer rising of at least four-fold should not be excluded from the list of suspected infecting serovars. On the other hand, low titers could indicate cross-reactivity between the serovars used in MAT [31, 72]. Accordingly, we cannot corroborate the susceptibility of horses to different local *Leptospira* serovars, since the authors detected anti-*Leptospira* antibodies by a single MAT, in addition to the fact that co-infections can only be confirmed by molecular typing tools [73]. Although the manuscript was unpublished at the time of the review, Orlando *et al*. [74] found 100% positivity in 108 horses from the coast, but samples were also analyzed by a single MAT using a panel of six serovars (Bataviae, Bratislava, Canicola, Grippotyphosa, Sejroe, and Tarassovi).

The reviewed studies reported a total of 16 *Leptospira* serovars in Ecuador based on antibody detection by MAT. This is considered the reference assay for diagnosing leptospirosis, and the information obtained on the infecting serovars is valuable from an epidemiologic standpoint. However, some intrinsic limitations of the MAT could lead to misinterpretations of the results obtained. Laboratories must include panels of *Leptospira* spp. with all locally circulating serovars because an incomplete panel should be responsible for false negative results [72, 75]. In previously un-surveyed areas, there is no guarantee that the panel is complete, and antibodies to unknown serovars may be missed in serological studies. In contrast, the identification and inclusion of new local serovars in the antigen MAT panels have revealed an increase in the prevalence of leptospirosis [76]. In cases, where serological tests are expected to be of poor sensitivity, incorporating molecular methods may be more appropriate. Molecular techniques can be used for leptospirosis surveillance and source tracking [73, 77]. We found no research identifying the serovars in the region, and a limited number of studies relied on a molecular diagnosis. Our research group was the first to report a pathogenic *Leptospira* species from the reproductive system of an asymptomatic boar by PCR and amplicon sequencing [43]. However, the authors also highlight that the need for bacteriological isolation of local serovars is critical for increasing the accuracy of MATs. Therefore, due to the limitations described above, the results provided in this review could suggest that there may be local leptospiral cycles that are not yet fully understood, and the results should be interpreted with great caution due to possible bias.

## Conclusion

The lack of studies linking the interrelationships between people, animals, and the environment, combined with the limitations of the serological techniques used, show that the epidemiology of *Leptospira* is not yet well established in Ecuador. Considering the country’s socio-economic challenges, future research should explore the use of sentinel species such as the horse, which would allow the development of longitudinal studies, increasing cost-efficiency surveillance. These studies should include a combination of serological and molecular diagnostic techniques to characterize the diversity of leptospira present in different bioclimatic regions of the country.

## Authors’ Contributions

EAD: Conceptualization, methodology, writing original draft, and editing. GA, CS, and LM: Methodology and data compilation. VB: Methodology, data compilation, writing review, and supervision. All authors have read, reviewed, and approved the final manuscript.
